# Supplementary material for: Interpretable machine learning and signal processing for automated reading and quality control of lateral flow tests for schistosomiasis
Source: Nat Commun. 2026 May 18;17:6515. doi: 10.1038/s41467-026-73094-0 (PMC13376430; doi:10.1038/s41467-026-73094-0)
Supplement: Supplementary file 3 — Reporting Summary [file 41467_2026_73094_MOESM3_ESM.pdf]

nature portfolio

Corresponding author(s): Goylette F. Chami

Last updated by author(s): Apr 21, 2026

Reporting Summary

Nature Portfolio wishes to improve the reproducibility of the work that we publish. This form provides structure for consistency and transparency in reporting. For further information on Nature Portfolio policies, see our Editorial Policies and the Editorial Policy Checklist.

Statistics

For all statistical analyses, confirm that the following items are present in the figure legend, table legend, main text, or Methods section.

n/a	Confirmed	
☐	☒	The exact sample size (*n*) for each experimental group/condition, given as a discrete number and unit of measurement
☐	☒	A statement on whether measurements were taken from distinct samples or whether the same sample was measured repeatedly
☐	☒	The statistical test(s) used AND whether they are one- or two-sided<br>*Only common tests should be described solely by name; describe more complex techniques in the Methods section.*
☐	☒	A description of all covariates tested
☐	☒	A description of any assumptions or corrections, such as tests of normality and adjustment for multiple comparisons
☐	☒	A full description of the statistical parameters including central tendency (e.g. means) or other basic estimates (e.g. regression coefficient) AND variation (e.g. standard deviation) or associated estimates of uncertainty (e.g. confidence intervals)
☐	☒	For null hypothesis testing, the test statistic (e.g. *F*, *t*, *r*) with confidence intervals, effect sizes, degrees of freedom and *P* value noted<br>*Give P values as exact values whenever suitable.*
☒	☐	For Bayesian analysis, information on the choice of priors and Markov chain Monte Carlo settings
☒	☐	For hierarchical and complex designs, identification of the appropriate level for tests and full reporting of outcomes
☐	☒	Estimates of effect sizes (e.g. Cohen's *d*, Pearson's *r*), indicating how they were calculated

Our web collection on statistics for biologists contains articles on many of the points above.

Software and code

Policy information about availability of computer code

Data collection	Open Data Kit (ODK) was used for data collection (ODK Collect application version 2022.4).
Data analysis	Python v3.9.6 with SciPy v1.11.3 and PeakUtils v1.3.4 for signal processing. OpenCV-Python v4.7.0 for image processing. MMDetection framework version 3.2.0 for image segmentation. ResNet-50, MobileNetV2, and ViT for image classification.<br><br>Code for the image classification pipeline is available via the University of Oxford research repository at https://doi.org/10.25446/oxford.32054937, while image segmentation code is restricted due to intellectual property held by Vanderbilt University.

For manuscripts utilizing custom algorithms or software that are central to the research but not yet described in published literature, software must be made available to editors and reviewers. We strongly encourage code deposition in a community repository (e.g. GitHub). See the Nature Portfolio guidelines for submitting code & software for further information.

Data

Policy information about availability of data

All manuscripts must include a data availability statement. This statement should provide the following information, where applicable:

- Accession codes, unique identifiers, or web links for publicly available datasets
- A description of any restrictions on data availability
- For clinical datasets or third party data, please ensure that the statement adheres to our policy

Image data are protected and are not publicly available due to data privacy laws, ethics approvals and participant agreements associated with the ongoing

SchistoTrack Cohort study.

Research involving human participants, their data, or biological material

Policy information about studies with human participants or human data. See also policy information about sex, gender (identity/presentation), and sexual orientation and race, ethnicity and racism.

Reporting on sex and gender	We did not include sex and gender in our analysis as we only used a diagnostic test result and its image for this study. Gender was self-reported and not disaggregated differently for the experiments in this study.
Reporting on race, ethnicity, or other socially relevant groupings	We have included information at the district level including the average air temperature in each district, as well as a statistic about differences between districts in the disagreement of results from two of our methods. We do not report any information at the village, tribe or religion level.
Population characteristics	See life sciences study design section below
Recruitment	We randomly sampled a total of 1952 households, from which 3853 individuals were recruited as clinical participants, from village registers on MDA records. All households with at least one child and one adult residing in the village for at least six months of the year were eligible. Data from 3188 of all clinical participants was available and used for this study. Written informed consent was obtained for adult participants, with adults consenting on behalf of the children after receiving their informed assent. Ethics approval was obtained from all local ethics committees. Participants were compensated in-kind for their time with a large (1kg) bar of soap.
Ethics oversight	Data collection and use were reviewed and approved by Oxford Tropical Research Ethics Committee (OxTREC 509-21), Vector Control Division Research Ethics Committee of the Uganda Ministry of Health (VCDREC146), and Uganda National Council of Science and Technology (UNCST HS 1664ES).

Note that full information on the approval of the study protocol must also be provided in the manuscript.

Field-specific reporting

Please select the one below that is the best fit for your research. If you are not sure, read the appropriate sections before making your selection.

[x] Life sciences [ ] Behavioural & social sciences [ ] Ecological, evolutionary & environmental sciences

For a reference copy of the document with all sections, see nature.com/documents/nr-reporting-summary-flat.pdf

Life sciences study design

All studies must disclose on these points even when the disclosure is negative.

Sample size	The sample size of this study was 3188, which is the number of study participants who provided a valid urine sample and did not have missing information for urine testing.
Data exclusions	No data was excluded.
Replication	The main outcome of urine diagnostic test was repeated up to three times until a valid result was obtained. Only the valid result was recorded.
Randomization	Not relevant for this study.
Blinding	There was no contamination between training and test data subsets for the machine learning methods used in the study.

Reporting for specific materials, systems and methods

We require information from authors about some types of materials, experimental systems and methods used in many studies. Here, indicate whether each material, system or method listed is relevant to your study. If you are not sure if a list item applies to your research, read the appropriate section before selecting a response.

Materials & experimental systems

n/a	Involved in the study
☒	☐ Antibodies
☒	☐ Eukaryotic cell lines
☒	☐ Palaeontology and archaeology
☒	☐ Animals and other organisms
☐	☒ Clinical data
☒	☐ Dual use research of concern
☒	☐ Plants

Methods

n/a	Involved in the study
☒	☐ ChIP-seq
☒	☐ Flow cytometry
☒	☐ MRI-based neuroimaging

Clinical data

Policy information about clinical studies

All manuscripts should comply with the ICMJE guidelines for publication of clinical research and a completed CONSORT checklist must be included with all submissions.

Clinical trial registration	Not applicable as this was an observational study.
Study protocol	Not applicable as this was an observational study.
Data collection	Data collection took place in January to February 2023 in Pakwach, Buliisa and Mayuge.
Outcomes	The primary outcome was diagnotic test results for schistosomiasis using image analysis methods on POC-CCA RDT images.

Plants

Seed stocks	Not applicable
Novel plant genotypes	Not applicable
Authentication	Not applicable
