# Supplementary material for: Interpretable machine learning and signal processing for automated reading and quality control of lateral flow tests for schistosomiasis
Source: Nat Commun. 2026 May 18;17:6515. doi: 10.1038/s41467-026-73094-0 (PMC13376430; doi:10.1038/s41467-026-73094-0)
Supplement: Supplementary file 2 — Peer Review file [file 41467_2026_73094_MOESM2_ESM.pdf]

nature portfolio

Peer Review File

Interpretable machine learning and signal processing for automated reading and quality control of lateral flow tests for schistosomiasis

Corresponding Author: Professor Goylette Chami

This file contains all reviewer reports in order by version, followed by all author rebuttals in order by version.

Attachments originally included by the reviewers as part of their assessment can be found at the end of this file.

Version 0:

Reviewer comments:

Reviewer #1

(Remarks to the Author)

(See attachment)

(Remarks on code availability)

Reviewer #2

(Remarks to the Author)
This study repeats previous work on the utility of the POC-CCA test in control programs for Schistosoma mansoni infections, but with the addition of machine learning applications to make decisions on test results. The authors assessed a large number of participants in a relatively high prevalence area of Uganda as part of a larger schistosomiasis cohort study. The results using image capture and machine learning were more aligned with supervisor readings of the POC-CCA cassettes than were the results as read by field technicians.

As new technologies are considered (in this case, ML), there is a balance between the advantages of the data accuracy provided by the new method and the costs of what it would take in time and training to ensure the new method is applied appropriately. Regardless of the reading method, field technicians will be the personnel capturing the results. Thus, potential errors in accuracy of visual POC-CCA readings would need to be balanced against the increased time and complexity to use a light box for image capture, uploading the data, and having someone sufficiently trained to manage the ML data output. This will presumably become easier with time but is likely still several years in the future. The question is, does the increased accuracy warrant the increased effort to employ the new system? If trace is considered negative as the authors suggest, the difference in sensitivity and specificity between ML and field readers is <1% and <10%, respectively. Perhaps a better use of an ML module would a training system for field technicians to make more accurate visual readings.

To do this, it would have been useful for the authors to show if treatment decisions would have been different in the implementation areas when comparing field technician readings to ML results, along with the relative costs in time and money of the two approaches. This would probably be quite difficult to demonstrate in this region of Uganda where prevalence is so high. Demonstrating a difference in program decision making based on reading method would be easier to demonstrate in a lower prevalence area. However, because this study was an add on to the ongoing SchistoTrack project, the authors unfortunately did not have this option. A future study in areas where the need for mass drug administration is less obvious a priori would perhaps be a better way to differentiate whether treatment decisions would be different when using visual readings and ML assisted readings.

The arguments the authors make for time saving by tests being able to be read earlier when using the ML system or a home

test and treat option are not really tenable. It is hard enough to ensure consistent quality application of a protocol without throwing in that sometimes a different read timing can be used and to extend this testing to untrained individuals, the test would need to be developed to a level where results are as clearcut as home pregnancy tests, which is extremely unlikely for this application.

Figure 1 presents the test-to-control ratio against reference standards in different laboratories. It would be helpful to add the positivity cutoff line at 0.15. But even without this as a reference, it appears that the antigen concentration needed for the test to become positive is 2-3 times higher in the Uganda lab than at Mondial or Vanderbilt, why is this?

Some other technical deficiencies in the manuscript that the authors should consider in any revisions are the failure to define a number of abbreviations on first mention (e.g., ASSURED and REASSURED--by the way, ASSURED criteria have been adopted, REASSURED are only proposed), the failure to define the number of stool and slides used to obtain Kato-Katz results and a presentation of these results for the study areas, and the labeling of the figures (e.g., figure 4--neither panel makes sense with the range of numbers used if they really represent log(EPG+1)).

(Remarks on code availability)

Version 1:

Reviewer comments:

Reviewer #1

(Remarks to the Author)
No further comments. The authors have done a great job revising the manuscript.

(Remarks on code availability)

Reviewer #2

(Remarks to the Author)
For the most part, the authors have answered the previous review concerns adequately. However, I am still a bit concerned about the data in Figure 1 showing that the antigen concentration required for a positive (as well as some higher signal ratios) is 2-3 fold higher in the endemic country laboratory than the antigen concentration in the industry or university lab. This argues that the pipeline thresholds will need to be recalibrated in the different field settings in which it will be used. I am worried that endemic country laboratories may not have staff with the expertise to do this. My guess is that visual reading of the cassettes will be the preferred method in most locations.

(Remarks on code availability)

Open Access This Peer Review File is licensed under a Creative Commons Attribution 4.0 International License, which permits use, sharing, adaptation, distribution and reproduction in any medium or format, as long as you give appropriate credit to the original author(s) and the source, provide a link to the Creative Commons license, and indicate if changes were made.
In cases where reviewers are anonymous, credit should be given to 'Anonymous Referee' and the source.
The images or other third party material in this Peer Review File are included in the article's Creative Commons license, unless indicated otherwise in a credit line to the material. If material is not included in the article's Creative Commons license and your intended use is not permitted by statutory regulation or exceeds the permitted use, you will need to obtain permission directly from the copyright holder.
To view a copy of this license, visit https://creativecommons.org/licenses/by/4.0/

REVIEWER #1

Reviewer 1, comment 1: Chris Ho and co-authors introduce an interpretable, automated image-processing pipeline integrating machine learning (ML) and signal-processing for reading lateral flow tests (LFTs), specifically targeting the point-of-care circulating cathodic antigen (POC-CCA) assay for Schistosoma mansoni. Using field-collected and laboratory-calibrated data, the authors benchmark their pipeline against visual readings from field and senior technicians, as well as commercial LFT readers. The work addresses an important global health challenge, especially in improving diagnostic accuracy and surveillance in low-resource settings. However, despite the novelty, several methodological, technical, and interpretive issues require attention to ensure robustness, generalizability, and reproducibility.
Author response: Thank you for recognising the novelty of the manuscript and for providing helpful suggestions on improving the clarity of the manuscript. We have incorporated your suggestions, which have improved the manuscript.

Reviewer 1, comment 2: The study involves a large dataset (n = 3,188 participants) from Uganda and integrates both technician-read and digitally processed test strips. Inclusion of calibrated antigen samples provides a valuable benchmark for test signal intensity. However, the manuscript lacks detail on participant selection criteria, demographics, infection intensity stratification, and quality control of field data acquisition. How were these 3,188 samples selected? Were they randomly sampled or from a specific surveillance program?
Author response: In the Methods, Section “Field technician readings and image capture”, Paragraph 2, we indicate that all participants within the SchistoTrack Cohort who attended the 2023 follow-up with one urine sample and one photo of the test formed the 3188 participants. We now add detail about how the SchistoTrack Cohort sampling (random sampling of households from communities) was conducted to the Methods, Section “Context and participant sampling”, Paragraph 1. “Sampling was conducted using uniform probability, with households assigned random numbers from one to the total number of households within each village.” Some information of demographics (age and sex) of the participants is now provided in the Results, Section “POC-CCA visual reading outcomes”, Paragraph 1 when we first mention how many participants were studied. “The mean age of the participants was 25.9 years (SD = 18.4), and 53.8% (1716/3188) were female.” We’re a bit unclear what is being referred to as infection intensity stratification as the paper is based on positive diagnosis and the line scan signal intensity cut-off was chosen from another paper to avoid arbitrarily fitting the cut-off to maximise infection diagnoses (which we investigated in detail in Figure S1 and the Results, Section “Image analysis outcomes for field images”, Paragraph 1 to provide evidence of the choice of cut-off). If you are referring to the supplementary analyses of infection intensity by eggs per gram (EPG), Supplementary Figures S10 (c) and S11 (b, d) in the Results Section “Influence of deviations from the commercially-suggested reading time”, Paragraph 2. Quality control is a central focus of this paper; we provide detailed information on the data acquisition including image characteristics (pixel resolution and image dimensions), configuration of the Open Data Kit survey and Android tablets used, procedures for following up missing results, and the level of training and experience of technicians conducting the tests. In addition, we describe standardised image capture protocols, including control of lighting conditions and object distance by a lightbox, consistent positioning and labelling of the cassette, and fixed Android tablet camera settings. These measures, which are used to ensure the quality of inputs to the machine learning models, are described in detail in the Methods, Section “Field technician readings and image capture”, Paragraph 2.

Reviewer 1, comment 3: Were repeated measurements or duplicate tests taken to control for batch variability?
Author response: Thank you; we also were worried about batch variability and that is why we used a single batch for all samples/participants and all districts. There is only one batch and the batch number has been provided in the Methods, Section “Sample and test preparation”, Paragraph 2. We now add a sentence to clarify “A single batch was used to control for batch variability.”

Reviewer 1, comment 4: What percentage of the tests failed or were considered invalid?

Author response: We have now added the following sentence to the Methods, Section "Field technician readings and image capture", Paragraph 1. **"**Approximately less than 0.5% of tests had to be repeated, largely due to the quality of the urine samples, where participants were asked to bring a new sample because of visible contamination in the urine pot due to menstruation or dirt."

Reviewer 1, comment 5: The environmental variability (light, humidity, cassette age) in field imaging and its control is not discussed, despite its known impact on colorimetric LFT interpretation.
Author response:
Fieldwork conditions, including seasonality (dry season), open-air set-up, and district-specific climatic conditions (temperature), are described in the Methods, Section "Context and participant sampling", Paragraph 1. The difference in disagreements between automated and field technician readings (when trace was considered negative) across the three districts was insignificant, as shown in the Results, Section "Agreement with visual readings of field images", Paragraph 2. High agreement between automated and field technician results despite different temperatures in the districts, suggesting robustness to environmental variability, is further discussed in the Discussion, Paragraph 2. The variations in lighting conditions were controlled with the use of a white plastic lightbox with the cassette placed in the centre and image captured through a hole opening in the lightbox, as described in the Methods, Section "Field technician readings and image capture", Paragraph 2. The cassette age and its variability captured by the different fieldwork periods in the three districts have now been added to the Methods, Section "Sample and test preparation", Paragraph 2. "The test cassettes were manufactured in November 2022, approximately two months before the start of fieldwork. Variability in cassette age over the course of the study would be captured by district-specific fieldwork periods (17–27 January in Pakwach, 29 January–5 February in Buliisa, and 11–16 February in Mayuge)." We have now added the lack of humidity control in our study as a limitation in the Discussion, Paragraph 2. "Humidity was not controlled in this study and may be a limitation, as relative humidity strongly affects liquid transport and reaction dynamics in paper-based lateral flow assays (Das, D. et al. (2022))."

Reviewer 1, comment 6: The study hinges on the comparison between "field technician", "senior technician", "linescan output", and "visual interpretation of calibrated references." However: The ground truth is ambiguous. While senior technician rereads are treated as a pseudo-gold standard, inherent subjectivity remains, especially for borderline trace readings.
Author response:
For validation of signal intensities, calibrated reference samples measured using a lateral flow reader were treated as the gold standard and used to validate the linescan pipeline. For classification tasks using the automated pipeline and deep learning models, senior technician re-readings were used as the reference standard due to their greater experience and readings being taken from images rather than direct observations. While we acknowledge that senior technician readings remain subjective, they represent the most consistent and experienced interpretation available under field conditions. The lack of a true gold standard for evaluating POC-CCA results makes objective comparison across different methods challenging. To address this, we conducted a comprehensive comparison across the different evaluation methods of the POC-CCA results in our study. We now explicitly acknowledge the absence of a true gold standard for POC-CCA evaluation in the Discussion, Paragraph 2. "Another limitation of this study is the absence of a true gold standard for interpreting POC-CCA results, making fully objective comparisons challenging. We therefore conducted a comprehensive comparison of the different POC-CCA evaluation methods, and used senior technician readings as the reference standard where applicable."

Reviewer 1, comment 7: Trace readings are inconsistently handled: sometimes considered positive, sometimes negative. While this is tested explicitly (e.g., Supplementary Figs S7-S8), the primary conclusion appears overly deterministic about traces being "better treated as negative." With the absence of a true gold standard for POC-CCA and clearly define how labeling was handled in final performance evaluations. Authors might consider including quantitative testing results using microscopy or PCR for at least a subset of samples.

Author response: We are a bit confused at where the inconsistency mentioned in how trace readings were handled. In your later comments (comment 10), you commend our work for systematically handling trace. We systematically present all results with trace treated as either all positive or as all negative. In later investigations, such as those in Supplementary Figures S2(c), S4(c) and S6(b), we present the range of signal intensities observed with trace results (remembering that signal intensities do not depend on any trace readings and that trace is a visual assessment), so that these signal intensities indicate what visual interpretation of trace means. Thank you for suggesting that we also bring in the microscopy to interpret the trace results. We have added to the Results, Section "Image analysis outcomes for field images", Paragraph 2, the following. "Consistent with these findings, KK microscopy classified 87.2% (455/522) of visual trace assignments as negative in participants with valid results."

Reviewer 1, comment 8: Multiple model architectures were tested (ResNet50, MobileNetV2, ViT, Linescan), which is appreciated, and the use of interpretable features (signal intensity ratios, region-based segmentation) adds credibility to the framework. However, the exact training data composition (size, label balance, augmentation) is not described. Were models trained on senior technician labels only? Were trace labels excluded or included? No cross- validation is mentioned. All metrics seem based on a fixed validation/test split.
Author response: Thank you for pointing out where clarifications are needed. We describe the total number of observations (which is equal to the number of participants as each have one image/sample) and label balance (negative, trace, positive) for every model in each split in the Methods, Section "Comparison to deep learning classification", Paragraph 1 and we have now added label balance in the splits for the different classification experiments in a new table, Supplementary Table S5. "For all experiments, the labeled datasets were split into training, validation, and test sets using a 70-15-15% stratified split, resulting in dataset sizes of 2230, 479, and 479 for the field-based labels, and 222, 48, and 48 for the senior technician labels. Supplementary Table S5 shows the class distribution across the stratified splits for both label sources." We trained models on field technician labels as well as senior technician labels and compared the two – for each experiment models were retrained from scratch. We now clarify this procedure in the Methods, Section "Comparison to deep learning classification", Paragraph 1 with the addition of the following sentence. "Models were trained on field technician labels as well as senior technician labels for comparison with the models retrained from scratch for each experiment." The results are presented in the Results, Section "Comparison to deep learning classification", Table 2. The table legend has been expanded to provide a clear overview of the different label sources and trace treatment strategies used across the classification experiments. Trace labels were included and also used systematically as done throughout the paper (one version of the model trained on trace as all negative and another trained on trace as all positive). We also performed a sensitivity analysis by training models with trace results excluded to evaluate the impact on classification. While this cleaner dataset yielded slightly better performance metrics, we have chosen to focus on treating trace results as either positive or negative, to adhere to the manufacturer's interpretation guidelines, which require every test to be classified as either positive or negative. To address the concern regarding lack of cross-validation, we have now included performance metrics from a 5-fold cross-validation approach in Supplementary Table S4. Specifically, while we kept an out-of-sample test dataset to ensure better generalisability, we resampled the training and validation splits five times. This allowed us to obtain five distinct models and calculate the mean and standard deviation of our performance metrics. This approach demonstrates that our model performance is stable and not dependent on a specific training/validation partition. We add all of this information now more clearly to our Methods, Section "Comparison to deep learning classification", Paragraph 2. "Moreover, 5-fold cross-validation was conducted to assess the generalisability of the deep learning models, with the test dataset kept constant and the remaining data resampled into five different training and validation datasets. The mean and standard deviation for sensitivity, specificity, and accuracy were calculated across the resulting five models." We have updated the Results, Section "Comparison to deep learning classification", Paragraph 1, to reflect this analysis. "Performance metrics for the 5-fold cross-validation of the deep learning classification experiments are provided in Supplementary Table S4. Standard deviations remained low for the field data metrics, ranging from 0.003 to 0.028, while higher values were observed for the senior technician data metrics due to the

smaller size of the dataset.” We have also added justification for a fixed test set to our Methods Section “Comparison to deep learning classification”, Paragraph 1. “The reason for the fixed test set that is never seen by the models during training and tuning is more similar to having an out-of-sample dataset or external dataset, which lends more support to the generalisability of the model by showing its performance on data it has never seen. All of our metrics are provided on the test dataset only.”

Reviewer 1, comment 9: There is limited discussion of overfitting, model robustness, or performance on out-of-distribution samples.
Author response: Thank you for your comments. We have addressed the concerns regarding overfitting by providing training metrics as described now in the Methods Section “Comparison to deep learning classification”, Paragraph 2, “The training and validation accuracies at each epoch were recorded to generate plots of training metrics for each model.” The training metric curves are added as Supplementary Figures S15 and S16, which are mentioned in the Results, Section “Comparison to deep learning classification”, Paragraph 1, “Training metrics for the field and senior readings dataset are shown in Supplementary Figures S15 and S16 respectively.” These curves show that validation and training accuracy follow a similar trajectory and remain closely aligned, indicating no evidence of overfitting. Moreover, the early stopping described in the Methods Section “Comparison to deep learning classification”, Paragraph 2, ensured that training was terminated when validation accuracy stopped improving substantially, thereby preventing overfitting. The stability and performance of our models across the different classification experiments with two cases of trace treatment further suggest the robustness of our models.
To address performance on out-of-distribution (OOD) samples, we have added analysis of 33 images from a reference library generated in 2025 featuring the POC-CCA3 test. This test has a differently shaped cassette than the tests used for model training. Our results show that the segmentation models successfully segmented the test membranes for this new cassette shape without retraining (mAP = 0.658). Moreover, when visual trace results were treated as negative, we observed a high agreement between the ViT model, the signal-processing pipeline and the technician. The ViT model achieved 100% sensitivity and 94.4% specificity against the linescan on these OOD samples. This high level of agreement suggests that our model has learned to isolate the diagnostic membrane from the test cassette, ensuring reliability across different cassette shapes. These analyses are described in the Methods, Section “Performance of image analysis pipeline on out-of-distribution cassette shapes”, as follows. “To evaluate the generalisability of our pipeline to out-of-distribution (OOD) samples, we utilised a reference library of 33 images collected in 2025. This dataset featured POC-CCA3 (Entezami, M. et al. (2025)) (Mondial Diagnostics) cassettes, which had a different shape compared to the cassettes used during the 2023 fieldwork. We assessed whether the two Mask R-CNN models with a ResNet-50-FPN0 and ResNeXt-101-32x8d-FPN backbones respectively, originally trained on 2023 data to only detect the membrane could segment the cassette membranes in these OOD sample images without retraining. Performance was quantified using the mAP with 95% CIs derived from 1000 bootstrap resamples. Finally, the best classification model trained on field images based on AUC performance was applied to the segmented POC-CCA3 images. To ensure consistency, we considered both versions of the best model, one trained on field images where trace was considered positive, and another where trace was considered negative. Its predictions were then compared against the automated signal processing pipeline and manual technician-assigned visual labels to assess diagnostic consistency.” The corresponding results are described in the Results, Section “Performance of image analysis pipeline on out-of-distribution cassette shapes”, as follows. “The image analysis pipeline demonstrated robustness to OOD samples without requiring model retraining. As shown in Supplementary Figure 18, the segmentation models accurately segmented the membranes of the differently shaped POC-CCA3 cassettes. The Mask R-CNN ResNet-50-FPN and ResNeXt-101-32x8d-FPN models achieved mAP values of 0.658 (95% CI: [0.597, 0.741]) and 0.676 (95% CI: [0.617, 0.756]), respectively. Classification of these OOD samples using the ViT model trained on field images showed strong alignment with linescan results (Supplementary Figure S19). Specifically, agreement was high when visual trace results were treated as negative. In this case, the ViT model showed high agreement with technician visual readings. Moreover, when using the linescan results as the reference standard, both the technician readings and the ViT classifications performed well.”

Reviewer 1, comment 10: The comparison between human and automated readings across different thresholds (trace = positive vs. trace = negative) is systematic and statistically sound. Specificity metrics for field *technicians* are alarmingly low (e.g., 51.6% vs. senior technician; Supplementary Fig S7). However, these figures are reported without discussion of false positive impact or implications for treatment decisions.
Author response: Thank you, we now add a new figure (Figure 4) to show how machine learning classifications may impact treatment decisions based on the infection prevalence of an area and agreement with field technicians as described in the Methods, Section "Evaluation of the image analysis pipeline", Paragraph 3, as follows. "To assess differences in treatment decisions, linescan classifications were further compared to field technician readings in different village-level prevalence setting categories (<30% for low, >=30 and <75% for moderate, and >=75% for high prevalence by POC-CCA; <10% for low, >=10 and <50% for moderate, and >=50% for high prevalence by KK) defined by the 2022 WHO guideline on control and elimination of human schistosomiasis (WHO guideline on control and elimination of human schistosomiasis 2022). Although WHO guidelines define the categories based on prevalence in school-aged children, we applied these thresholds to our full study population which included both children and adults." Results have been added to the Results Section "Agreement with visual readings of field images", Paragraph 3 as follows. "We also evaluated the percentage of individuals who would have had or not had treatment based on the difference in positive cases from linescans and field readings in different prevalence settings. In low-prevalence settings, linescan classification would have identified 13.0% fewer individuals (9/69) as needing treatment compared to field technician readings when considering trace as positive, and 1.2% (3/245) fewer when considering trace as negative (Figure 4). In moderate-prevalence settings, linescans classified 22.1% (418/1894) fewer individuals as positive when trace results were considered positive, and 4.9% (129/2615) fewer when trace was considered negative. In high-prevalence settings, linescan classification would have resulted in 22.2% (272/1225) fewer individuals being treated when trace was considered positive, and 11.6% (38/328) fewer when trace was considered negative. In settings stratified by KK prevalence (Supplementary Figure S9), linescan classification similarly identified fewer individuals for treatment across all prevalence settings." We have also added a few sentences about the implications of the low specificity for increasing costs to national programmes and undermining surveillance efforts for verifying areas for elimination, to the Discussion, Paragraph 9, as follows. "Moreover, the high rate of false positives in field readings leads to significant over-treatment, increasing the financial burden on national programs and risking overestimation of prevalence levels. Our findings from treatment decision assessments highlight the potential for automated classification to improve diagnostic specificity for surveillance and optimise drug distribution. However, future implementation studies must evaluate the cost-effectiveness of these treatment decisions in the context of both mass drug administration efforts and primary healthcare centres."

Reviewer 1, comment 11: Model metrics (sensitivity, specificity, accuracy) are provided, but no ROC curves, AUC values, or confidence intervals for key comparisons are shown in the main text.
Author response: Thank you for these suggestions. We have now computed ROC curves and calculated the AUCs for all classification experiments as described in the Methods, Section "Comparison to deep learning classification", Paragraph 1, "To evaluate the discriminative power of the classifiers across all probability thresholds, we generated Receiver Operating Characteristic (ROC) curves and calculated the Area Under the Curve (AUC)." We have included these to complement the single-threshold metrics (sensitivity, specificity, and accuracy) provided in the main text. The results are presented in Supplementary Figure S15 (ROC curves) and Supplementary Table S3 (AUCs with 95% CIs). These are mentioned in the Results, Section "Comparison to deep learning classification", Paragraph 1, as follows. "The AUC values for the classifiers ranged from 0.899 to 0.991 (Supplementary Table S3). The corresponding ROC curves are provided in Figure S15." We also clarify that the information in brackets in Table 2 are the confidence intervals and were constructed via bootstrap methods. This information is now noted in the legend.

Reviewer 1, comment 12, Main figures should be numbered and included in the text. Figure legends are often minimal; more detail will help readers to interpret Figures effectively. For example, Figure 2 lacks information on axes and classifier types. Colors should be explained on the figure as well.
Author response: We have double checked that all main and supplementary figures are numbered and confirmed they are all numbered and cited in the main text. We have expanded the legend to explain the axes and colours as follows. "(a) Overall distribution (grey), (b) Distribution by positive (pink), trace (green), and negative (blue), (c) Distribution by positive (pink) and negative (blue) where trace is considered positive, and (d) Distribution when trace is considered negative." We also have updated the legends of Supplementary Figures S3 and S14 to clarify similar information.

Reviewer 1, comment 13: Suppl. Figures S13 – S14 provide excellent insights into test line appearance dynamics, these could be partially migrated into the main paper.
Author response: Thank you; we have moved Supplementary Figure S13 to the main text and renumbered the main text and supplementary figures accordingly.

Reviewer 1, comment 14: While overall writing is clear, some overclaims exist (e.g., "solves visual trace ambiguity" is too strong).
Author response: Thank you – we have removed this phrase and also added a discussion about trace decisions, especially the differences when compared to if field technician labels are used or, as we still advocate, for senior technician labels to be used. We have added the following to the Discussion Section, Paragraph 8, "While the automated approach may not resolve all ambiguity in diagnosis, it mitigates variability in trace visual assignments, ensuring reproducible results."

Reviewer 1, comment 15: Clarify abbreviations early (e.g., LFT, POC-CCA, KK). Use ml or mL through the entire manuscript.
Author response: Thank you, we have clarified the abbreviations now on first use throughout the manuscript and use mL throughout.

Reviewer 1, comment 16: It is recommended to include discussion of similar tools such as smartphone LFT readers, or WHO-endorsed semi-quantitative scales for schistosomiasis diagnosis. Please discuss why POC-CCA remains imperfect despite these advancements, and why LFA is inherently non-quantitative, and how ML/DL tools are used to partially address this challenge.
Author response:
We thank the reviewer for suggesting the inclusion of these comparisons. We have updated the Discussion, Paragraph 7, to address these points. "We have demonstrated the ability to train technicians under difficult field circumstances and time constraints to use simple lightboxes to upload images suitable for ML pipelines. However, to make our pipeline usable without the addition of any setup or training of someone to run analysis scripts, it would benefit from future work to develop a software application such as a smartphone LFT reader. Such a tool could automatically adjust differences in lighting to remove the need for a lightbox while providing real-time outputs from our pipeline on test and quality control results without the need for expertise to run the pretrained ML models.

Despite advancements in digital diagnostic tools, POC-CCA remains imperfect for schistosomiasis surveillance since model training still relies on subjective visual labels rather than calibrated samples. Moreover, test limitations such as batch-to-batch variability and a lack of a quantitative scale due to poor correspondence with microscopy impact the consistency of results. Our findings suggest that ML tools can partially address these challenges by providing a standardised interpretation, establishing a pathway toward more objective diagnostics."

REVIEWER #2

Reviewer 2, comment 1: This study repeats previous work on the utility of the POC-CCA test in control programs for *Schistosoma mansoni* infections, but with the addition of machine learning applications to make decisions on test results. The authors assessed a large number of participants in a relatively high prevalence area of Uganda as part of a larger schistosomiasis cohort study. The results using image capture and machine learning were more aligned with supervisor readings of the POC-CCA cassettes than were the results as read by field technicians.
Author response: We thank the reviewer for the suggestions made to improve the manuscript; all requests have been comprehensively addressed and have improved the application of the manuscript. To clarify, as noted in the introduction, the purpose of the study was to build an automated pipeline for lateral flow tests for schistosomiasis rather than to focus on implementation studies of POC-CCA utility as was done more generally in the SCORE consortium with a comparison to KK microscopy. To our knowledge, there exist no automated pipelines for schistosomiasis lateral flow readings nor a way to automate incorporation of quality control. We also directly address a call for a recent review in *Nature Comms (Han et al. 2025 https://doi.org/10.1038/s41467-025-58527-6)* to improve automated pipelines for point-of-care diagnostics, moving beyond visual readings alone without reference libraries and extending the testing of the pipelines to real-world settings with variable conditions. Hence, the focus of our study on the machine learning. Now that we have provided a generalisable and validated machine learning pipeline – comparing two drastically different methods of machine learning classification and signal processing, we have enabled future work that can be focused on implementation studies. As such, we do not see our study repeating any previous work done.

Reviewer 2, comment 2: As new technologies are considered (in this case, ML), there is a balance between the advantages of the data accuracy provided by the new method and the costs of what it would take in time and training to ensure the new method is applied appropriately. Regardless of the reading method, field technicians will be the personnel capturing the results. Thus, potential errors in accuracy of visual POC-CCA readings would need to be balanced against the increased time and complexity to use a light box for image capture, uploading the data, and having someone sufficiently trained to manage the ML data output. This will presumably become easier with time but is likely still several years in the future. The question is, does the increased accuracy warrant the increased effort to employ the new system?
Author response: Thank you for the comment. We have added a couple of sentences in the Discussion, Paragraph 7, about considerations for future use. "We have demonstrated the ability to train technicians under difficult field circumstances and time constraints to use simple lightboxes to upload images suitable for machine learning pipelines. However, to make our pipeline usable without the addition of any setup or training of someone to run analysis scripts, it would benefit from future work to develop a software application such as a smartphone LFT reader. Such a tool could automatically adjust differences in lighting to remove the need for a lightbox while providing real-time outputs from our pipeline on test and quality control results without the need for expertise to run the pretrained machine learning models."

You are right in that field technicians would still be responsible for image capture. We believe all that is required to make our new technology immediately usable is the development of a software application that can be attached to a standard phone or tablet. In this system, a lightbox would not be needed to control for the light settings in the field. However, we would like to emphasize that we validated our protocols in complex field settings where intense fieldwork was ongoing and technicians were part of a larger team with several other clinical responsibilities. The conditions were challenging, working directly in the villages with variable temperatures and some as extreme as 40C. We provide extensive detail on the lightbox and setup of the cassettes for reading, and cannot anticipate why this would add other unanticipated complexity. An alternative to a lightbox could be a simple cardboard box, pasted with white paper, and with a camera flash used to control lighting. The uploading of the data should not pose complications as we used a standard tablet, but a phone with a camera could be used as a replacement given all that was done for data upload was to take the photo within an Open Data Kit (ODK) Survey. ODK is already the mainstay for the World Bank, WHO,

NGOs, and policymakers conducting evaluation studies or collecting primary data. No expertise is even required to make these surveys as ODK offers a user interface or many alternatives exist for using ODK (e.g. Enketo). No expertise is required to use these surveys beyond a tap of a camera button and click of save. For the most complicated part of the technology, there is no need for someone to be trained in the machine learning pipeline, as the models are pre-trained now and could be used to classify new images simply uploaded from a phone or tablet. This validation was done by having a hold-out dataset of images the full machine learning pipeline did not see during training. An upload feature would be the first part of a software application development, which is underway already at Vanderbilt University by Tom Scherr's lab.

Reviewer 2, comment 3: If trace is considered negative as the authors suggest, the difference in sensitivity and specificity between ML and field readers is <1% and <10%, respectively.
Author response: Thank you for this comment. While the difference in sensitivity and specificity between ML and field readers reduces when trace is considered negative, the key strength of the ML pipeline is the elimination of human subjectivity. Unlike field readers, whose interpretations of trace results vary across settings and individuals, the ML pipeline applies a consistent classification threshold, ensuring that results are reproducible.

Reviewer 2, comment 4: Perhaps a better use of an ML module would a training system for field technicians to make more accurate visual readings.
Author response: Thank you for this excellent suggestion for future work. We have added this suggestion to the Discussion, Paragraph 7. "We have only evaluated our pipeline for its ability to provide diagnoses and assess the quality of the test. Future work is needed to assess how these results could be used to train new technicians, potentially having the technicians first classify the cassette and then the outputs from the pipeline provide the suggested trace classification, diagnosis, and quality assessment for the new technicians to use to review images and practice further classifying additional tests. This application may be a more direct use of our pipeline than the provision of diagnoses, and may save costs for national control programmes by reducing the need to hire expert technicians to conduct extensive training and review of new technicians."

Reviewer 2, comment 5: To do this, it would have been useful for the authors to show if treatment decisions would have been different in the implementation areas when comparing field technician readings to ML results, along with the relative costs in time and money of the two approaches. This would probably be quite difficult to demonstrate in this region of Uganda where prevalence is so high. Demonstrating a difference in program decision making based on reading method would be easier to demonstrate in a lower prevalence area. However, because this study was an add on to the ongoing SchistoTrack project, the authors unfortunately did not have this option. A future study in areas where the need for mass drug administration is less obvious a priori would perhaps be a better way to differentiate whether treatment decisions would be different when using visual readings and ML assisted readings.
Author response: Thank you for this suggestion, which we have incorporated in the revision. SchistoTrack villages have diverse prevalence, ranging from 5.8-93.5% as measured by POC-CCA in the year that we did this study. We have added the following to the Results, Section "POC-CCA visual reading outcomes", Paragraph 1, about village-level prevalences. "The village-level prevalences (calculated across 52 villages, with 8 smaller adjacent villages grouped into 4 pairs for a total of n=48 village units) ranged from 18.8-93.5% (median 71.9%, IQR 58.8-79.7, n=48 village units) when trace was considered positive, and 5.8-84.1% (median 52.3%, IQR 39.8-66.2, n=48 village units) when trace was considered negative." We have generated confusion matrices in a new figure, Figure 4 and added it to the main manuscript to show differences in diagnoses that would have been made based on the prevalence of the village. We provide figure panels for trace negative and trace positive and divide each panel into WHO village prevalence categories as provided in the WHO schistosomiasis guidelines from 2022 (low, moderate, high). Each panel shows the confusion matrix, which is the percent of individuals who would have had or not had praziquantel treatment based on the difference in positive cases. Here we show that by using ML-based classification, we would have classified 13% less individuals as needing treatment than using field technician readings (considering

trace results as positive) alone in a low-prevalence setting. In a high-prevalence setting, the ML method would have classified 22.2% fewer individuals needing treatment compared to field readings when trace is considered positive. These results have been added in a short paragraph that focuses on treatment decisions in the Results, Section “Agreement with visual readings of field images”, Paragraph 3. “We also evaluated the percentage of individuals who would have had or not had treatment based on the difference in positive cases from linescans and field readings in different prevalence settings. In low-prevalence settings, linescan classification would have identified 13.0% fewer individuals (9/69) as needing treatment compared to field technician readings when considering trace as positive, and 1.2% (3/245) fewer when considering trace as negative (Figure 4). In moderate-prevalence settings, linescans classified 22.1% (418/1894) fewer individuals as positive when trace results were considered positive, and 4.9% (129/2615) fewer when trace was considered negative. In high-prevalence settings, linescan classification would have resulted in 22.2% (272/1225) fewer individuals being treated when trace was considered positive, and 11.6% (38/328) fewer when trace was considered negative. In settings stratified by KK prevalence (Supplementary Figure S9), linescan classification similarly identified fewer individuals for treatment across all prevalence settings.” We also have added sentences to the discussion where we focused on the future use of our results for implementation studies and indicated the need to cost out what these changes in treatment decisions would have for both mass drug administration programmes and primary health care centres. “Our findings from treatment decision assessments highlight the potential for automated classification to improve diagnostic specificity for surveillance and optimise drug distribution. However, future implementation studies must evaluate the cost-effectiveness of these treatment decisions in the context of both mass drug administration efforts and primary healthcare centres.”

Reviewer 2, comment 6: The arguments the authors make for time saving by tests being able to be read earlier when using the ML system or a home test and treat option are not really tenable. It is hard enough to ensure consistent quality application of a protocol without throwing in that sometimes a different read timing can be used and to extend this testing to untrained individuals, the test would need to be developed to a level where results are as clearcut as home pregnancy tests, which is extremely unlikely for this application.
Author response: Thank you for the comment. We’re not sure about the difficulty of the application of the time saving component without further testing. We have now added a sentence in the Discussion, Paragraph 9 to indicate that “Further testing is required to see if the time-saving component would apply to at-home test and treat strategies for untrained individuals or mainly in research and clinical settings with trained personnel.” For the clearer test result, our pipeline is applicable to other lateral flow tests developed for schistosomiasis or even other pathogens using urine samples, so with improved diagnostics like the rapid diagnostic test for circulating anodic antigens, which is underway (and mentioned in the discussion), then clearer results would be possible and trace results would be less ambiguous. More broadly, machine learning pipelines (see review Han et al 2025) for diagnostics frequently demonstrate time saving and require some protocol with no published evidence elsewhere of these procedures not being tenable.

Reviewer 2, comment 7: Figure 1 presents the test-to-control ratio against reference standards in different laboratories. It would be helpful to add the positivity cutoff line at 0.15. But even without this as a reference, it appears that the antigen concentration needed for the test to become positive is 2-3 times higher in the Uganda lab than at Mondial or Vanderbilt, why is this?
Author response: Thank you. We have added the cutoff line to Figure 1. The higher antigen concentration required for a positive result in the Uganda laboratory may be due to variation in imaging conditions, such as lighting, and environmental factors like humidity or storage conditions, that can influence the lateral flow assay and detection of the test line signal by the pipeline.

Reviewer 1, comment 8: Some other technical deficiencies in the manuscript that the authors should consider in any revisions are the failure to define a number of abbreviations on first mention (e.g., ASSURED and REASSURED--by the way, ASSURED criteria have been adopted, REASSURED are only proposed), the failure to define the number of stool and slides used to obtain Kato-Katz results

and a presentation of these results for the study areas, and the labelling of the figures (e.g., figure 4--neither panel makes sense with the range of numbers used if they really represent log(EPG+1)).
Author response: Thank you for pointing out these clarifications. We have defined the REASSURED acronym now on first use and ensure to avoid noting any official WHO endorsement, though the criteria are based on the WHO ASSURED criteria and are widely used in modern diagnostics given the number of citations from companies, NGOs, and academics of the original paper (https://www.nature.com/articles/s41564-018-0295-3). In the Methods, Section "Assessment of signal intensities over time", Paragraph 1, we now clearly state that "The KK microscopy results were obtained from one stool sample with duplicate thick smear slides read by two independent technicians, including a further 10% of slides randomly sampled for rereading by a senior technician for quality control." Thank you for spotting the mistake in the x-axis labels for Figure 4, these have been corrected by flipping the figure. We checked all figures in the main text and supplement again carefully to ensure no other mistakes were present.

REVIEWER #1

Reviewer #1 (Remarks to the Author):
No further comments. The authors have done a great job revising the manuscript.

Author response: We thank the reviewer for their positive feedback and for their support of our revised manuscript.

REVIEWER #2

Reviewer #2 (Remarks to the Author):
For the most part, the authors have answered the previous review concerns adequately. However, I am still a bit concerned about the data in Figure 1 showing that the antigen concentration required for a positive (as well as some higher signal ratios) is 2-3 fold higher in the endemic country laboratory than the antigen concentration in the industry or university lab. This argues that the pipeline thresholds will need to be recalibrated in the different field settings in which it will be used. I am worried that endemic country laboratories may not have staff with the expertise to do this. My guess is that visual reading of the cassettes will be the preferred method in most locations.

Author response: We thank the reviewer for this insightful observation regarding the requirement of a higher antigen concentration for a positive result in the endemic country laboratory. We agree with the reviewer that requiring field staff to recalibrate the pipeline for different settings is not viable. The variation observed in our results highlights the impact of environmental factors and imaging conditions on the lateral flow tests. However, we do not advocate for site-specific recalibration of thresholds. Instead, our findings suggest the need for further validation studies across different contexts to establish either a universal threshold or a defined range of acceptable thresholds that maintain robust diagnostic performance. Future research is essential to determine how measurement errors associated with the threshold impact prevalence estimates, which in turn would affect policy decisions. We have now added the following in the Discussion, Paragraph 7. "However, realising this potential requires ensuring the pipeline is robust across different settings. To avoid the need for site-specific recalibration, future research is required to establish a universal pipeline threshold or an acceptable range of thresholds across different contexts. It is important to understand how measurement errors around these thresholds impact prevalence estimates, which are essential for national programmes to inform policy decisions on whether to target populations for mass drug administration or to monitor transmission."

ROUND 1 REVIEWER 1 ATTACHMENT:

Summary

Chris Ho and co-authors introduce an interpretable, automated image-processing pipeline integrating machine learning (ML) and signal-processing for reading lateral flow tests (LFTs), specifically targeting the point-of-care circulating cathodic antigen (POC-CCA) assay for *Schistosoma mansoni*. Using field-collected and laboratory-calibrated data, the authors benchmark their pipeline against visual readings from field and senior technicians, as well as commercial LFT readers.

The work addresses an important global health challenge, especially in improving diagnostic accuracy and surveillance in low-resource settings. However, despite the novelty, several methodological, technical, and interpretive issues require attention to ensure robustness, generalizability, and reproducibility.

Major comments

- The study involves a large dataset (n = 3,188 participants) from Uganda and integrates both technician-read and digitally processed test strips. Inclusion of calibrated antigen samples provides a valuable benchmark for test signal intensity. However, the manuscript lacks detail on participant selection criteria, demographics, infection intensity stratification, and quality control of field data acquisition. How were these 3,188 samples selected? Were they randomly sampled or from a specific surveillance program? Were repeated measurements or duplicate tests taken to control for batch variability? What

percentage of the tests failed or were considered invalid? The environmental variability (light, humidity, cassette age) in field imaging and its control is not discussed, despite its known impact on colorimetric LFT interpretation.

- The study hinges on the comparison between “field technician”, “senior technician”, “linescan output”, and “visual interpretation of calibrated references.” However: The ground truth is ambiguous. While senior technician rereads are treated as a pseudo-gold standard, inherent subjectivity remains, especially for borderline trace readings. Trace readings are inconsistently handled: sometimes considered positive, sometimes negative. While this is tested explicitly (e.g., Supplementary Figs S7-S8), the primary conclusion appears overly deterministic about traces being “better treated as negative.” With the absence of a true gold standard for POC-CCA and clearly define how labeling was handled in final performance evaluations. Authors might consider including quantitative testing results using microscopy or PCR for at least a subset of samples.
- Multiple model architectures were tested (ResNet50, MobileNetV2, ViT, Linescan), which is appreciated, and the use of interpretable features (signal intensity ratios, region-based segmentation) adds credibility to the framework. However, the exact training data composition (size, label balance, augmentation) is not described. Were models trained on senior technician labels only? Were trace labels excluded or included? No cross-validation is mentioned. All metrics seem based on a fixed validation/test split. There is limited discussion of overfitting, model robustness, or performance on out-of-distribution samples.
- The comparison between human and automated readings across different thresholds (trace = positive vs. trace = negative) is systematic and statistically sound. Specificity

metrics for field technicians are alarmingly low (e.g., 51.6% vs. senior technician; Supplementary Fig S7). However, these figures are reported without discussion of false positive impact or implications for treatment decisions. Model metrics (sensitivity, specificity, accuracy) are provided, but no ROC curves, AUC values, or confidence intervals for key comparisons are shown in the main text.

Minor weaknesses

- Main figures should be numbered and included in the text. Figure legends are often minimal; more detail will help readers to interpret Figures effectively. For example, Figure 2 lacks information on axes and classifier types. Colors should be explained on the figure as well. Suppl. Figures S13 – S14 provide excellent insights into test line appearance dynamics, these could be partially migrated into the main paper.
- While overall writing is clear, some overclaims exist (e.g., “solves visual trace ambiguity” is too strong). Clarify abbreviations early (e.g., LFT, POC-CCA, KK). Use ml or mL through the entire manuscript.
- It is recommended to include discussion of similar tools such as smartphone LFT readers, or WHO-endorsed semi-quantitative scales for schistosomiasis diagnosis. Please discuss why POC-CCA remains imperfect despite these advancements, and why LFA is inherently non-quantitative, and how ML/DL tools are used to partially address this challenge.
